# Guidelines for sharing individual level patient data

**DOI:** 10.17179/excli2026-9817

**Published:** 2026-10-05

**Authors:** Agapios Sachinidis, Wiebke Albrecht, Jan G. Hengstler

**Affiliations:** 1 Center for Physiology, Faculty of Medicine and University Hospital Cologne, University of Cologne, Robert-Koch-Str. 39, 50931 Cologne, Germany; 2 Department of Toxicology, Leibniz Research Centre for Working Environment and Human Factors (IfADo), Ardeystr. 67, 44139 Dortmund, Germany; 3 Department of Toxicology, Faculty of Chemistry, TU Dortmund, 44227 Dortmund, Germany

## Abstract

EXCLI Journal has long encouraged authors to make anonymized individual-level data available as supplementary material whenever this can be done responsibly. Recent discussions, however, have raised concerns about whether even anonymized laboratory data from individual patients may create unacceptable privacy risks. We examine this question from both a scientific and a practical perspective. We first consider why underlying data should be preserved and shared: they can support verification of published results, allow alternative analyses, and preserve information for future research. We then stress-test the privacy argument by constructing hypothetical scenarios in which a published table of individual laboratory values could lead to identification of a participant, disclosure of previously unknown health information, and concrete harm. These scenarios show that such a chain of events is possible, but that its likelihood depends strongly on the characteristics of the dataset and on information already available to a potential attacker. Small datasets containing rare, distinctive, or potentially stigmatizing findings require particular caution. Based on this analysis and the existing literature, we propose the following principles for EXCLI Journal: appropriately anonymized individual-level clinical measurements and data derived from biological samples should generally be shared when the residual risk is low and the scientific value is substantial. More sensitive or distinctive datasets may require aggregation, removal of potentially identifying variables, or controlled access. Authors invoking institutional restrictions on public data sharing should provide the relevant policy to the editors, and such restrictions should not prevent editors and reviewer from accessing the underlying data for verification. Our aim is not to eliminate every conceivable risk, but to establish a proportionate approach that protects participants while preserving valuable data for future science.

## ⁯⁯⁯

### Why do we ask for the underlying data?

Recent events have prompted us to discuss with our authors and readers how we should deal with the data underlying the manuscripts submitted to EXCLI Journal. We see two different reasons for making these data available. The first is the integrity of the published article. Editors and reviewers sometimes encounter data patterns that raise questions about the plausibility of a study. For example, variances or control values may differ unexpectedly from those seen in comparable experiments. In most cases, such findings have a simple and legitimate explanation. However, we have also encountered rare cases in which we had serious reason to suspect that a study was based on fabricated data.

This raises a dilemma that we think should be made explicit. The overwhelming majority of scientists work carefully and honestly. A request from an editor to provide the underlying data may nevertheless be perceived as an expression of distrust: Why should authors who have submitted a manuscript be asked to prove that their data are genuine? We do not want our authors to be treated as suspects simply because they submit a manuscript. At the same time, we believe that it would be wrong to ignore the fact that fabricated manuscripts do occur, and that they are not necessarily easy to recognize. We have seen manuscripts in which the research question was clear and relevant, the figures were internally consistent, and the conclusions appeared to follow from the results. Only closer examination revealed small features that raised concerns. Such features are not, in themselves, evidence of fabrication. They are warning signs that may justify asking further questions.

This creates a difficult balance for editors. We have a responsibility to identify problematic studies and to reduce the risk that fabricated research enters the scientific literature. At the same time, we should not create an editorial process in which honest scientists are routinely treated as if they were under suspicion. We therefore need ways of checking questionable results that are proportionate, transparent, and reasonable for both authors and editors. Asking for the data underlying a manuscript can be one such way. Researchers who have conducted their work properly will usually have their technical and biological replicate data, or the measurements from individual experimental subjects, available in suitable formats such as Excel or CEL files. Providing these data to the editors or reviewers therefore usually requires little additional work. If the data have already been prepared for this purpose, making them available as an electronic supplement to the published article may also be a straightforward step. Access to the underlying data can thus provide editors with a practical way to check the plausibility of questionable results without creating a disproportionate burden for authors.

### Preserving data means facilitating future discoveries

The second reason for making the data underlying a publication available is scientific. Individual measurements contain information that is not captured by the summary values usually presented in a paper. They allow other researchers to examine the distribution of observations, assess variability, compare their own results with those of previous studies, and perform alternative analyses. This can be particularly valuable when addressing a new research question. A researcher may initially generate only a small number of new datasets. One of the first questions is then whether these observations are representative or whether they happen to reflect an unusual sample. This question is much easier to answer if the individual data from previous studies are available. Instead of comparing one new dataset with a few published means or summary statistics, researchers can place their observations in the context of many previously studied individuals and examine how closely the new data resemble the existing range and distribution. In this way, previously published individual data can serve as a reference against which new observations can be evaluated.

There is, however, a fundamental problem: in many areas of biomedical research, the data underlying a publication are not preserved in a form that remains available for future research. Once the study has been published, the individual observations may remain only in the laboratory files of the researchers or institution. Over time, files may be lost, software and storage systems may change, researchers may leave their institutions, and data that were never deposited in a durable repository may eventually become inaccessible. When this happens, the scientific information contained in the individual observations is lost, even though the publication itself remains available. This loss is more than a technical inconvenience. A study is designed to answer a particular question, but the individual observations may later become valuable for questions that could not have been anticipated when the study was performed. They may allow comparison with a new patient population, contribute to a larger dataset, or be reanalyzed using methods that were not available or considered at the time. Once the underlying data have disappeared, these possibilities cannot be recovered.

For this reason, we believe that preserving the data underlying published research deserves to be considered part of preserving the scientific record itself. A publication preserves today's conclusions; the data may enable tomorrow's discoveries.

### Which data are we talking about? From experimental replicates to individual patients

Before discussing data availability, it is useful to clarify what we mean by the data underlying a publication. The term “raw data” can refer to quite different types of information.

First, there are the individual measurements from technical replicates from which means, curves, or other summary results are calculated. Second, there are measurements from individual biological units, for example individual animals in an animal experiment. For both types of data, we see no reason not to make them available to editors and readers. They are an integral part of the experimental results and are generally not associated with concerns about the privacy of individual persons.

A third category is different: measurements from individual human participants. A typical example is a table containing the laboratory values of individual patients or volunteers, together with a limited number of characteristics such as age or sex, but without names or other direct identifiers. Such data can be scientifically particularly valuable. They preserve information about individual observations that may be useful for verification, comparison with other studies, and future research. Ultimately, making such information available may contribute to scientific progress far beyond the question that motivated the original study.

At the same time, individual data from human participants require particular care. They concern real people, and the fact that data have been deidentified does not by itself settle the question of whether they should be made publicly available. This is not merely a theoretical issue for us. In recent months, three groups whose manuscripts were under consideration informed us that they were not permitted to provide individual patient data because of internal institutional rules. We found ourselves in a difficult position: such restrictions may reflect legitimate requirements for the protection of research participants, but they also prevent editors from examining the underlying data when questions arise and may prevent the scientific community from benefiting from these data in the future.

This tension is the focus of the following discussion. How much additional risk to individual participants is actually created by making appropriately deidentified laboratory data available, and how should that risk be weighed against the scientific value of preserving and sharing the data?

### Can an individual laboratory result actually harm a patient?

The question we therefore need to ask is more specific than whether individual laboratory data can theoretically be linked to a person. We should ask:

Can the publication of a typical table of individual laboratory values accepted by EXCLI Journal, under realistic conditions, provide new information about a particular person, make that person identifiable, and thereby cause a concrete disadvantage?

To take this question seriously, we tried to construct such situations ourselves. We deliberately looked for worst-case scenarios. In doing so, we sometimes had to make demanding assumptions and imagine unusually determined actors with questionable intentions trying to extract as much information as possible from a published dataset. This may sound somewhat artificial. However, if we are to argue for making individual-level data available, we should be prepared to test that position against deliberately unfavorable assumptions. We therefore asked what a particularly determined “bad actor” could actually do with a table of individual laboratory results.

The usual argument for restricting access to individual health data is easy to understand. Individual laboratory results are health data. Even if names and other direct identifiers are removed, several characteristics may sometimes be combined to identify a person. Once identified, information about that person's health could potentially be used against them, for example by an insurer, an employer, or another person. This sounds plausible. But how would this work with an actual EXCLI table?

Imagine a clinical study in which patients receive either placebo or compound X. The paper reports glucose, HbA1c, and a new laboratory parameter for each participant, together with sex and year of birth:


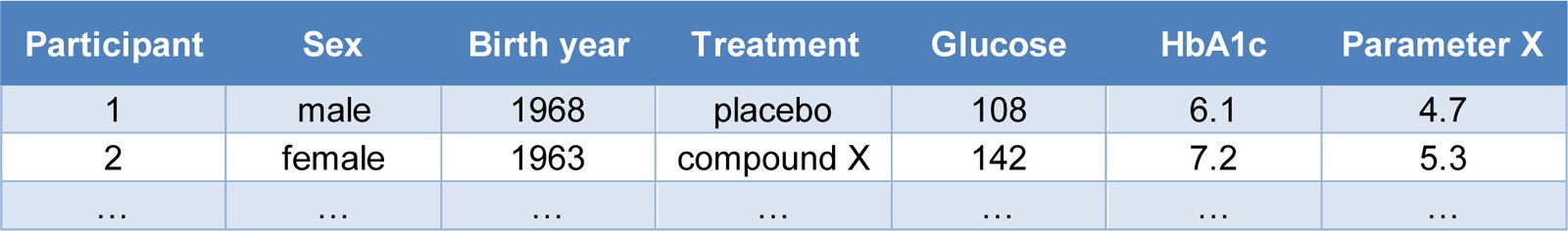


There are - of course - no names, full dates of birth, addresses, insurance numbers, DNA or RNA sequences, or other obvious direct identifiers. Now imagine someone who wants to use the data to cause harm. The first problem is surprisingly simple: they have to determine which row belongs to which person.

The cases we considered are described in Box 1. We deliberately chose situations in which the person attempting to cause harm has as much prior information as reasonably possible. We considered an insurer who knows that a particular person participated in the study, an employer who knows about the participation, and a person who already knows the participant personally. We also considered a more extreme situation in which the published findings are so rare and distinctive that they may themselves point to a particular individual.

These scenarios helped us to identify the conditions that would have to come together for publication of an individual laboratory result to cause a concrete disadvantage. First, the person must be identifiable from the published information, either alone or in combination with information already available to them. Second, the publication must provide genuinely new information about that person. Finally, that information must be capable of causing a realistic disadvantage.

The first three cases show that such a chain of events is theoretically possible, but also how difficult it is to complete the chain from publication to identification, new information, and finally concrete harm. For a typical table of individual laboratory values, this appears to be a highly unlikely sequence of events. The fourth case is different. When a study involves very few participants, rare or highly distinctive findings, or information that may carry a risk of stigma or social disadvantage, the published data themselves may make identification substantially easier. Such situations require a more cautious approach.

The question is therefore not whether individual laboratory data can ever create a privacy risk. Theoretically, they can. The more relevant question for editorial practice is how often this risk is realistically created by the type of individual laboratory data that we encounter in biomedical publications, and whether that risk is sufficiently small to be outweighed by the scientific value of making the data available.

### What have others concluded?

Our thought experiment (Box 1) raises the question if our considerations correspond to what others have concluded about the sharing of individual patient data. The literature does not provide a simple answer. There is broad agreement that individual participant data can have substantial scientific value, but there is also broad recognition that such data should not necessarily be made freely available without considering the possibility of re-identification and harm.

El Emam et al. (2015[1]) described several important reasons for sharing individual patient data, including the possibility of verifying published findings, conducting new analyses, combining datasets, and making better use of data that have already been collected. At the same time, they emphasized that individual patient data need to be prepared for sharing in a way that reduces the risk of identification. Thus, the basic idea is not that individual-level data should either be shared or protected. Rather, the challenge is to obtain the scientific benefits of data sharing while keeping the associated privacy risks acceptably low. A similar position was reached by an international group working on the sharing and reuse of individual participant data from clinical trials. Ohmann et al. (2017[5]) proposed that individual participant data should become a normal part of research data sharing, but that the data should be appropriately de-identified and that different forms of access should be used according to the sensitivity of the data. Their guiding principle was that access should be “as open as possible and as closed as necessary.” Importantly, their recommendations do not treat all individual participant data as equally sensitive. Instead, they envisage a range of access models, including controlled access, depending on the characteristics of the dataset and the risk of misuse.

This is also consistent with more recent work on re-identification. Haber et al. (2022[3]), reviewing methods for anonymising structured tabular phenotype data, pointed out that simply removing names or other direct identifiers is not always sufficient to assess the risk of re-identification. The relevant question is how the remaining characteristics of a dataset can be combined and whether particular individuals can thereby be distinguished. This supports the approach taken in our thought experiment: the presence or absence of names is not, by itself, a sufficient test. What matters is what can actually be inferred from the information that remains. At the same time, the actual risk should not be confused with the mere possibility of re-identification. Ford et al. (2025[2]) recently examined this issue in the context of de-identified clinical free text, which in many respects contains considerably more potentially identifying information than a conventional table of laboratory values. They developed a framework for assessing re-identification risk and reviewed reported re-identification methods and safeguards. Their conclusion was that, when clinical text is appropriately de-identified and stored in secure data environments, the risk of patient re-identification is very low. This does not demonstrate that every dataset is safe, but it illustrates an important point: the practical risk depends not only on the theoretical possibility of identifying someone, but also on the type of data, the information available to a potential attacker, and the conditions under which the data can be accessed.

Rare diseases provide an important counterexample. Hansson et al. (2016[4]) discussed the particular difficulty of protecting anonymity in rare disease research. When only a small number of patients have a particular condition, information about the disease itself, together with age, sex, clinical features, or other characteristics, may make an individual recognizable. At the same time, the authors point out that sharing information between researchers is particularly important in rare diseases because the number of affected individuals is small. Rare disease research therefore illustrates especially well the tension between scientific value and privacy protection that we encountered in our fourth case.

Perhaps the most convincing evidence that individual-level health data can be scientifically valuable while still being protected comes from large research infrastructures. The UK Biobank is a particularly striking example. It contains data from approximately 500,000 participants, including questionnaire information, physical measurements, biomarkers, genetic data, imaging and linked health records (Sudlow et al., 2015[6]). These data have been used to investigate a very wide range of diseases and risk factors. The resource has also enabled studies involving highly sensitive information, including alcohol consumption and alcohol-related liver disease. For example, Whitfield et al. (2022[7]) used UK Biobank data on alcohol consumption, physiological measurements, laboratory results, hospital diagnoses and mortality to investigate mortality risks among participants with very high alcohol consumption. The continued use of such data in large-scale research reflects a broad scientific and societal acceptance of the principle that detailed individual-level health information can be made available for research when appropriate safeguards are in place. It also reflects the substantial scientific value attributed to preserving and reusing such data.

The UK Biobank example is also important for another reason. It does not simply place the complete individual-level dataset on the open internet. Personal identifying information is removed, and access to the research data is provided to approved researchers through a controlled research environment. Such controls clearly reduce the risk, but they cannot eliminate it completely. An approved researcher may misuse the access, information may be combined with other sources, or access may be obtained or used inappropriately. In other words, even this highly controlled system accepts some residual risk in order to make the scientific value of the data available. This reflects a broad consensus that the scientific value of detailed individual-level health data can justify accepting a very low residual privacy risk when appropriate safeguards are in place.

### How EXCLI Journal will handle individual-level patient data

This brings us back to the question relevant to EXCLI Journal. The literature does not suggest that individual-level health data are harmless simply because direct identifiers have been removed. Nor does it suggest that such data should generally be withheld. Instead, the prevailing approach is risk-based: the characteristics of the dataset, the possibility of identification, the information already available to a potential attacker, the sensitivity of the information that could be learned, and the conditions under which the data are accessed all need to be considered. This is also close to the distinction that emerged from our own four hypothetical cases. Large datasets containing common laboratory measurements are different from small datasets containing rare diseases, highly distinctive clinical findings, unusual phenotypes, or information that may expose individuals to stigma or social disadvantage. The scientific community has therefore developed a range of approaches rather than a single rule, from open access to controlled access and, in particularly sensitive situations, more extensive anonymisation or restriction of individual-level data.

For EXCLI Journal, this leads us to a research-friendly but risk-aware approach. We do not want privacy concerns to become a general reason for withholding individual-level laboratory data that can be shared responsibly. At the same time, we do not want to ignore situations in which publication could create a realistic risk to individual participants. Our starting point will therefore be that appropriately anonymised individual-level data should be made available when the residual risk is low and the scientific value of sharing the data is substantial. In practical terms, we will consider the following points when individual patient data are involved:

- **Routine individual laboratory data:** Individual measurements from sufficiently large studies, particularly common laboratory values or newly investigated biomarkers without distinctive clinical characteristics, should generally be made available as supplementary data when they have been appropriately anonymised.
- **Small or distinctive datasets:** Greater caution will be required when the number of participants is small or when the dataset contains rare diseases, rare genetic findings, unusual phenotypes, distinctive combinations of clinical characteristics, or information that could expose participants to stigma or social disadvantage. In such cases, variables may need to be removed or aggregated, access may need to be restricted, or publication of individual-level data may not be appropriate.
- **The possibility of identification:** The absence of names or other direct identifiers will not by itself be regarded as sufficient. Editors will consider whether a person with reasonable prior knowledge could identify an individual participant from the information provided.
- **Editorial and peer-review access:** Regardless of whether the individual-level data can ultimately be published, editors and reviewers should be able to examine the underlying data when this is necessary to assess the validity and plausibility of the manuscript.

There is one situation that requires particular clarification. Occasionally, authors may tell us that institutional rules prevent them from making appropriately anonymised individual-level data publicly available. We recognize that institutions may have legitimate policies governing the use and dissemination of patient data. However, we do not think that a general reference to an institutional rule should by itself be sufficient to prevent the scientific community from accessing data that can otherwise be shared responsibly.

We would therefore regard such restrictions as an exception rather than the rule. If authors rely on an institutional policy to withhold individual-level data from public access, we would ask them to provide the relevant policy or regulation to the editors. This would allow us to assess whether the restriction is specific, applicable to the dataset in question, and proportionate to the potential privacy risk. Importantly, such a restriction would not remove the requirement to provide the underlying data to the editors and reviewers when they are needed to evaluate the manuscript. Institutional restrictions on public data sharing should not become restrictions on editorial verification.

Our aim is therefore not to establish a rule of unrestricted data sharing, nor one of general data withholding. We want to establish a proportionate system in which individual laboratory and clinical data can remain available for science, while genuinely sensitive situations receive the specific additional protection they require. Scientific progress and participant protection should not be treated as opposing goals: the right approach is to protect people without unnecessarily losing the data that science may need tomorrow.

We do not regard these guidelines as a final or immutable set of rules. They are intended as a starting point for a policy that can evolve with scientific practice, experience, and new evidence. We therefore invite our authors and readers to share their experience and if necessary challenge these guidelines, provide realistic counterexamples, and help us refine the balance between scientific openness and participant protection.

**Box 1:** Hypothetical cases illustrating when publication of individual-level laboratory or clinical data may be acceptable or problematic

***Case 1: The insurer***

Consider an insurance company. One might argue that an insurer could use published HbA1c values to discover previously unknown diabetes among its policyholders. But this is not yet a mechanism. We have to follow the information step by step.

The hypothetical insurer would need to know:

1. that a particular person had participated in the study;
2. which row in the table belonged to that person; and
3. that the laboratory result in that row could reliably be attributed to that person.

The publication itself does not provide this link. An insurer that has access only to the EXCLI table sees, for example: female, born in 1963, HbA1c 7.2. It does not thereby know which of its policyholders this person is. To establish the link, it would need additional information about the study participants from another source. If obtaining such information required unauthorized access to the patient records of the participating institution, we would have to distinguish between the risk created by publishing the table and the risk created by the separate unlawful acquisition of information. The latter should not simply be attributed to the publication itself. So our hypothetical insurer has reached its first obstacle. The published table contains a health-related measurement, but it does not tell the insurer to whom that measurement belongs.

But let us make the situation more difficult for ourselves. Suppose that the insurer already knows that one of its customers participated in the study. Can it now identify the patient's row in the published table? Suppose, for example, that the insurer knows that one of its female customers, born in 1963, participated in the study and received compound X. If the published table contains several women born in 1963 who received compound X, the insurer may still not be able to determine which row belongs to its customer. The additional laboratory values do not necessarily solve this problem.

Now let us make the case even more favorable to the attacker. Imagine that there is only one participant in the table who is female, was born in 1963, and received compound X. The insurer can now reasonably assume that this is its customer. The publication has potentially created a link between a particular person and the laboratory results in that row.

At this point, however, we should ask another question: What information is actually new?

If the insurer already knew the patient's laboratory values from another source, the publication has merely confirmed information that was already available. The situation is different if the insurer knew only that the patient had participated in the study and that she had received compound X. If the published table now reveals that this particular person had an HbA1c of 7.2, the publication has provided genuinely new health information. We have therefore reached the basic mechanism we were looking for:

publication → identification → new health information → potential disadvantage.

This is a real privacy risk. It would be wrong to dismiss it simply because the names and other direct identifiers had been removed. But the example also shows that the risk does not arise simply because individual laboratory values have been published. Several conditions have to come together. The attacker must already have some information about the participant. The characteristics published in the paper must be sufficiently specific to identify the corresponding row. The laboratory result must provide genuinely new information about that person. And there must finally be a realistic way in which this information could cause a disadvantage.

How often are these conditions actually met by the type of individual laboratory data that we are considering? Our hypothetical insurer already had to know that the patient had participated in the study. This is not impossible. Patients may mention their participation to their insurer, employer, colleagues, or family members. In some studies, participation may also become known through other legitimate sources. We should therefore not regard this assumption as unrealistic simply because it is inconvenient for our argument.

The more difficult question is whether the information published in the paper would be sufficient to identify the patient's row. In our example, the insurer knew the patient's sex, year of birth, and treatment group. If the table contained 100 participants, there might be several rows with exactly these characteristics. Even adding ordinary laboratory values may not make one row unique. The attacker would then know that one of several rows belongs to the patient, but would not know which one.

Let us therefore make the situation even more favorable to the attacker. Suppose that the combination of sex, year of birth, treatment group, and laboratory values occurs only once in the table. The patient can now be identified with reasonable confidence.

But how unusual would such a situation actually be?

This depends strongly on the size and structure of the study and on the information included in the table. A small study with many characteristics can easily produce unique combinations. A larger study containing only broadly shared characteristics is much less likely to do so. The same laboratory value that is highly identifying in a study of 20 participants may be almost useless for identifying one person in a study of 2,000 participants.

There is another important point. Laboratory values are not generally unique identifiers in the way that a name, telephone number, or genetic sequence may be. Many people have similar glucose levels, HbA1c values, blood counts, liver enzymes, or other routine laboratory measurements. A combination of several values may become more distinctive, but the question is always whether it becomes distinctive enough to establish a reliable link to one particular person.

We should also ask what information the attacker would need to begin this search. If an insurer simply receives the published table without knowing who participated in the study, the problem is very different from the situation in which it already knows that a particular customer participated. In the first case, the table contains many possible individuals but no obvious way of connecting them to the insurer's customer database. In the second case, the attacker has already solved a substantial part of the identification problem. This distinction matters because it changes the role of the publication. The publication may contain the health information, but it may not contain the information needed to connect that health information to a particular person.

Finally, even successful identification would not automatically mean harm. Suppose the insurer correctly identifies a customer and learns from the paper that her HbA1c was 7.2 at the time of the study. What could the insurer actually do with this information? Would it be medically meaningful enough to indicate a previously unknown disease? Would the information still be current? Would it have any effect on the person's insurance status? And, most importantly, would the publication have caused a disadvantage that would otherwise not have occurred? These questions are not meant to suggest that such risks are irrelevant. They are meant to define the risk more precisely.

The strongest version of the argument is therefore not that anonymized individual laboratory data can never cause harm. Clearly, situations can be constructed in which identification and harm are possible. The more interesting question is whether those situations are sufficiently plausible and sufficiently common to justify a general restriction on publishing appropriately deidentified individual laboratory data. This leads us to a more demanding test. Instead of asking whether a harmful scenario can be imagined, we should ask whether a harmful scenario can be constructed that remains realistic when applied to the ordinary type of individual laboratory data published in a biomedical research article.

***Case 2: The employer***

Why do we need a second example? The insurance scenario has shown that a privacy risk can exist, but only when several unlikely conditions come together. It would be easy to conclude that the risk is small simply because an insurer normally does not know who participated in a particular study. But perhaps this is too favorable an assumption. We should therefore choose an attacker for whom knowledge of study participation is more plausible. A hypothetical employer provides such a case.

Imagine a study conducted among employees of a company, perhaps in cooperation with an occupational health service. The employer knows that some of its employees participated. It therefore does not have to discover this information from the published paper. Let us again give our hypothetical attacker the most favorable reasonable starting position: the employer knows that a particular employee participated in the study.

Now consider the same type of published table. It contains sex, year of birth, treatment group, glucose, HbA1c, and another laboratory parameter, but no name, address, or other direct identifier.

Could the employer identify the employee?

 If the study includes many employees of similar age and sex, probably not. Knowing that an employee participated still does not tell the employer which row belongs to that person. But let us again make the case as difficult as possible. Suppose the employee is the only woman born in 1963 who received compound X. The employer can now reasonably assign one particular row to her. The publication has therefore potentially revealed a new piece of health information: her HbA1c, or perhaps another abnormal laboratory value.

At this point, however, the same question arises as before: what can the employer actually do with this information? If the laboratory result indicates a possible health problem, could it influence the employee's career, promotion, assignment to a particular task, or continued employment? In principle, this is conceivable. An employer could have an interest in knowing whether an employee has a health condition that might affect work performance or fitness for a particular job. But again, we should not stop at the first plausible story. We have to follow the chain all the way to the alleged harm.

The employer must first identify the correct employee. The published characteristics must be sufficiently specific to distinguish that employee from all other participants. The laboratory result must provide genuinely new information rather than merely confirm something already known. The result must be sufficiently reliable and clinically meaningful to support an inference about the employee's health. And finally, the employer must be able and willing to use that information in a way that disadvantages the employee.

This last step is particularly important. A laboratory value in a research paper is not necessarily equivalent to a clinical diagnosis. A single HbA1c measurement, for example, does not by itself establish the complete medical status of a person. Even when the value is abnormal, its interpretation depends on the circumstances in which it was measured, the study protocol, the timing, and other clinical information.

Nevertheless, the employer example is more difficult to dismiss than the insurance example. The attacker may already know the identity of a participant, and in a study conducted within a workplace the connection between the participant and the study may be particularly easy to establish. We therefore have to acknowledge that a real risk can arise when a published dataset contains enough additional information to distinguish one participant from the others.

But this second example also teaches us something important. The decisive issue is not simply who the potential attacker is. The decisive issue is the amount of information that must be combined before the publication produces a new person-specific fact. In both examples, the same sequence is required:

knowledge of participation → identification of the individual row → new health information → realistic possibility of harm.

The examples therefore do not show that publishing individual laboratory data is harmless. They show something more precise: the privacy risk depends on the information available before publication, the information contained in the publication, and the information that can realistically be inferred from combining the two.

This is why hypothetical worst-case scenarios are useful. They help us move beyond the general statement that “health data are sensitive” and ask a more concrete question: What exactly does the publication make possible that was not possible before?

If we can construct realistic cases in which the answer is “something harmful,” those cases should influence how such data are handled. If we repeatedly need highly unusual combinations of circumstances to create such a case, that should also be part of the discussion.

***Case 3: The person who already knows the patient***

The insurance and employer examples still leave one possible objection. Perhaps we are making the attacker's task unnecessarily difficult because we assume that the attacker has to identify the participant from relatively little information. What if the attacker already knows the person very well? Consider a family member, close acquaintance, neighbour, or colleague who knows that a particular person participated in a study. Such a person may also know the person's approximate age, sex, medical history, and perhaps some laboratory results from conversations or previous medical encounters. This is a much stronger starting position than that of an unknown insurer or employer. Let us therefore make the case as difficult as possible.

Imagine that a patient's relative knows that she participated in a clinical study. The relative also knows that she was born in 1963 and received compound X. The published table contains exactly one woman born in 1963 who received compound X. The relative can therefore identify her row with reasonable confidence. The table now shows that her HbA1c was 7.2 and that another laboratory parameter was clearly outside the usual range.

Here, the chain we have been looking for is complete. The person was already known. The participant can be identified. The publication provides health-related information that may not previously have been known to the relative. A real privacy interest therefore exists.

But even this case deserves a closer look. Suppose the relative did not know the HbA1c value before reading the paper. The publication has now provided genuinely new information. Could this information cause harm?

Certainly, one can imagine situations in which it might. A family relationship could be affected by knowledge of a previously undisclosed disease. A colleague might react differently to a perceived health problem. A neighbour might use the information in a hurtful way. In the case of a public or socially exposed person, even a relatively minor health finding could become embarrassing or damaging if it were disclosed more widely.

At this point, however, we should distinguish two different questions. The first is whether the publication has made the health information accessible to someone who should not have had it. In our example, the answer may be yes. The second is whether this risk is created by the publication of an ordinary table of individual laboratory values or by the particular circumstances that we have deliberately constructed around it.

In our example, we have assumed that the relative already knew the study, knew that the person had participated, knew her sex and year of birth, knew the treatment group, and encountered a table in which these characteristics happened to identify a single participant. We have therefore given the attacker an unusually large amount of prior knowledge and, at the same time, a dataset in which the relevant characteristics happen to be highly distinctive.

This does not make the scenario irrelevant. On the contrary, it shows that even a relatively ordinary-looking table can become sensitive when combined with sufficient background knowledge. But it also suggests that the risk cannot be assessed from the published laboratory values alone. The same table may pose almost no identification problem for a reader who knows nothing about the participants, while posing a genuine privacy risk to someone who already knows a great deal about one of them.

There is another important observation. If the relative already knows that the person participated in the study and knows enough about her characteristics to identify the corresponding row, the publication has not revealed the person's existence in the study. It has only added the information contained in that row. The amount of new information therefore depends on what was already known.

This may seem like a subtle distinction, but it is central to our question. Privacy risk is not simply a property of the dataset. It arises from the combination of the dataset with the information and knowledge available to the person who receives it.

The social case therefore gives us the strongest warning so far. Unlike the hypothetical insurer, the attacker may plausibly know the participant personally. Unlike the employer, the attacker may also possess detailed background information that makes identification easier. We can therefore construct a realistic pathway from publication to identification and from identification to new health information.

At the same time, the case shows why the existence of such a pathway does not by itself tell us how much protection is required for every dataset. The relevant question remains how distinctive the published information is, how much prior knowledge an attacker would realistically have, and whether the resulting information could actually cause harm.

This is where the nature of the underlying data becomes decisive. A table containing common laboratory measurements from a sufficiently large and diverse study is fundamentally different from a table containing a rare disease, an unusual combination of findings, a very small number of participants, or highly distinctive genetic or longitudinal information.

The strongest privacy arguments may therefore concern not individual laboratory data as a category, but particular datasets whose characteristics make identification and harmful inference realistically possible.

***Case 4: The rare and highly distinctive finding***

So far, our examples have involved relatively common laboratory measurements. But perhaps this is where our reasoning reaches its real limit. What happens when the laboratory finding itself is rare and highly distinctive?

Consider a hypothetical study of a very rare genetic variant. The variant is associated with a recognizable physical phenotype, and the study investigates whether carriers also show a particular behavioural characteristic. The study includes only a small number of participants. Suppose the authors publish a table containing, for each participant, age, sex, treatment or study group, several laboratory measurements, and the genetic finding. Even if names and other direct identifiers are removed, one participant may be identifiable from the combination of the published characteristics alone.

The situation becomes even more problematic if the phenotype is recognizable outside the study. A person who knows one of the participants may be able to identify the corresponding individual without needing access to any confidential database. Once the person has been identified, the table may reveal information about a genetic condition and about a potentially stigmatizing behavioural association. This is a genuine privacy problem. The publication does not merely contain information that could theoretically be linked to a person. The information itself may be so distinctive that the link is readily apparent.

Such a case also illustrates an important difference between different types of individual data. A glucose value of 108 mg/dL is shared by many people and is therefore usually a weak identifier. A rare genetic variant associated with a recognizable phenotype may be almost the opposite: the finding can itself function as an identifier.

We would therefore not regard such a dataset as an ordinary example of the individual laboratory data that we are considering. If a study involved a very small number of participants, a rare genetic condition, a highly unusual phenotype, or a potentially stigmatizing association, we would expect the authors and editors to recognize that the data require special protection. Depending on the circumstances, this might mean aggregation, removal of particularly identifying variables, controlled rather than unrestricted access, or not publishing individual-level data at all.

This example is nevertheless important for our discussion because it demonstrates that our argument has a boundary. We are not claiming that deidentified individual data are always safe to publish. They are not. The more useful question is whether the same concern should apply to the much more ordinary situation in which a sufficiently large study reports individual values of common laboratory parameters, together with a small number of broad characteristics such as sex and age or birth year.

The rare-disease example gives us a clear answer to one part of the question: there are specific datasets for which individual publication can create a substantial and foreseeable privacy risk. But it does not tell us how large that risk is for ordinary individual laboratory data. In fact, it suggests that the two situations should not automatically be treated as equivalent.

## Declaration

### Conflict of interest

The authors are editors of EXCLI Journal, JGH is editor of Arch Toxicol. They declare no competing financial interests.

### Artificial Intelligence (AI) - assisted technology

No AI tools were used in the preparation of this editorial.

## References

[1] El Emam K, Rodgers S, Malin B (2015). Anonymising and sharing individual patient data. BMJ.

[2] Ford E, Pillinger S, Stewart R, Jones K, Roberts A, Casey A (2025). What is the patient re-identification risk from using de-identified clinical free text data for health research?. AI Ethics.

[3] Haber AC, Sax U, Prasser F, NFDI4Health Consortium (2022). Open tools for quantitative anonymization of tabular phenotype data: literature review. Brief Bioinform.

[4] Hansson MG, Lochmüller H, Riess O, Schaefer F, Orth M, Rubinstein Y (2016). The risk of re-identification versus the need to identify individuals in rare disease research. Eur J Hum Genet.

[5] Ohmann C, Banzi R, Canham S, Battaglia S, Matei M, Ariyo C (2017). Sharing and reuse of individual participant data from clinical trials: principles and recommendations. BMJ Open.

[6] Sudlow C, Gallacher J, Allen N, Beral V, Burton P, Danesh J (2015). UK Biobank: an open access resource for identifying the causes of a wide range of complex diseases of middle and old age. PLoS Med.

[7] Whitfield JB, Seth D, Morgan TR, GenomALC Consortium (2022). All-cause and liver-related mortality risk factors in excessive drinkers: Analysis of data from the UK Biobank. Alcohol Clin Exp Res.

